# Translation, Cultural Adaptation, Validity, and Internal Consistency of the Greek Version of the Individual Workload Perceptions Scale-Revised

**DOI:** 10.7759/cureus.19174

**Published:** 2021-10-31

**Authors:** Victoria Alikari, Evangelos C Fradelos

**Affiliations:** 1 Department of Nursing, University of West Attica, Athens, GRC; 2 Department of Nursing, University of Thessaly, Larissa, GRC

**Keywords:** individual workload perceptions scale, intent to stay, peer support, manager support, workload, nursing work environment

## Abstract

Introduction: The working environment in hospitals has been characterized as very important for the improvement of the provided care and the nurses’ job satisfaction. The aim of the current study was translation and cultural adaptation of the Individual Workload Perceptions Scale-Revised (IWPS-R) as well as the investigation of the validity and internal consistency of the scale.

Methods: This is a cross-sectional, descriptive study involving 365 Greek nurses from two large hospitals in Athens, Greece. Nurses completed the Individual Workload Perceptions Scale-Revised, which is a self-administered questionnaire consisting of 29 items on a five-point Likert-type scale. For the translation, the scale was first translated into the Greek language (forward translation) and then into the English language (backward translation) and culturally adapted. For the study of the construct validity, exploratory and confirmatory factor analyses were performed, while the criterion of the convergent validity was between the five factors of the scale. To study the reliability, the method of test-retest was performed while Cronbach’s alpha coefficient was used to study the internal consistency of the scale. Data analysis was performed via the Statistical Package for the Social Sciences (SPSS), version 21.0 (IBM Corp., Armonk, NY).

Results: According to the exploratory factor analysis, the Greek version of the Individual Workload Perceptions Scale-Revised consists of five factors (Manager Support, Peer Support, Unit Support, Workload, and Intent to Stay) explaining 51.4% of the total variance. From the confirmatory factor analysis, the model was equivalent to the original factorial structure of the IWPS-R. Τhe convergent validity revealed a positive correlation between all the domains of the scale (p < 0.001). The test-retest method showed that there are no significant differences between the first and the second measurements (intraclass correlation coefficient [ICC] = 0.990, p < 0.001). The internal consistency was very good (Cronbach’s alpha coefficient = 0.878).

Conclusions: The IWPS-R is a reliable and valid instrument for Greek nurses to measure the perceptions of the nursing working environment.

## Introduction

Nurses are among the most heavily stressed workers among the healthcare professionals due to the high workload and the constant high demands. The nursing work environment is one of the most important indicators for evaluating the safety and quality of care and is closely and strongly related to the quality of services provided to patients [1]. A healthy nursing work environment is accompanied by job satisfaction reducing absenteeism from work and withdrawal of nurses from the hospital [2].

A major factor that negatively affects the nursing work environment is the workload. The workload can be categorized as physical or mental. The mental workload is related to the processing of information in a short period. Nurses use their mental abilities to communicate with patients and their families, assess the disease, perceive any changes in the health status of patients, and intervene and prevent any deterioration [3]. Many nurses work in rotating shifts, which is a combination of day and night shifts and includes eight-hour or 12-hour shifts that alternate between the morning and night [4]. Nurses adapt to cyclical working hours, communicate, and discuss with colleagues about patients. The variety of problems that arise during the shift, the constant changes of thinking about the various tasks of the department, and the differences from patient to patient exceed the requirement for simple nursing care [5]. Unfortunately, all these mental and physical requirements that are necessary for the practice of nursing are ignored by the managers [6].

Therefore, nurses, as healthcare professionals, need a supportive work environment, which maximizes the effectiveness in treating diseases, the quality of care, nurses’ well-being, and job satisfaction [7]. Many characteristics of the work environment affect nurses’ job satisfaction. Organizational factors include organizational structure, policies and procedures, management systems, and working conditions [8]. Social factors include peer relationships, workgroups, and opportunities for interaction [9]. In the context of social interaction, the phenomenon of role conflict is very common, in which other individuals or groups with whom nurses interact have conflicting views or expectations. A common type of role conflict is role overload, in which many expectations are addressed to a nurse at a time. The conflict of roles is a result of the fact that nurses are forced to engage in activities other than nursing due to the lack of nursing staff and secretarial support. The performance of non-nursing duties in combination with the negatively charged work environment leads to increased levels of work-related stress and disorders such as chronic fatigue syndrome and burnout. As a result, nurses’ satisfaction and intent to stay at work are affected [10,11].

Understanding the complexity of nurses' work as well as the mental workload are prerequisites for patients’ safety. Based on the above, the focus of nursing research is on an effort to understand the perceived nursing work environment. Psychometric tools are used to measure the perceived nursing work environment, especially in magnet hospitals. The most commonly used questionnaires to measure the perceived nursing work environment is the original Individual Workload Perception Scale (IWPS) [12] consisting of 46 items, the Individual Workload Perceptions Scale-Revised (IWPS-R) [13] consisting of 29 items, the Nursing Work Index [14], and the Perceived Nursing Work Environment instrument [15]. The above instruments emphasize the nursing work environment and its dimensions such as the workload, management style, support from peers and managers, perceived productivity, and positive scheduling climate. In Greece, the Nursing Activity Score has been validated as a first attempt to measure the nursing workload [16]. It refers to categories of duties performed by nurses, but it does not emphasize the support by managers or peers, workload, and job satisfaction. For this reason, the researchers chose the IWPS-R as it is short in comparison to the original version and studies the above domains of the nursing work environment.

Based on the above, this study aimed to translate and culturally adapt the IWPS-R for Greek nurses as well as to test the reliability, construct validity, convergent validity, and internal consistency of this scale.

## Materials and methods

This is a cross-sectional study among 365 nurses. Nurses were selected from a general (G.N.A. G.Gennimatas Hospital of Athens) and a psychiatric hospital (Psychiatric Hospital of Athens Dafni) (convenience sample) in the broad area of Athens (the capital of Greece). Among the 521 nurses of the hospitals, 365 accepted to participate in this study (response rate, 70%). The inclusion criteria were as follows: the participants should be (i) permanent nurses (not temporary) and (ii) nurses for at least one year. Students of nursing were excluded from the study. Τhe final sample was based on the literature according to which 10 participants are required for each question for the studies of the reliability and validity of the scales [17]. The questionnaires were distributed by the researchers. Data were collected during the period between May 2021 and June 2021.

The instrument

The development of the IWPS-R [13] was founded on Maslow's theory of basic needs according to which the satisfaction of basic needs (sleep and lunch breaks) is a prerequisite for the satisfaction of higher needs (career development) [18]. The original version of IWPS was developed in 2003 [12] consisting of 46 items, while in 2010, the revised version consisting of 29 items was emerged [13]. The IWPS-R is a self-completed questionnaire rated on a five-point Likert-type scale (1 = totally disagree to 5 = totally agree) and five dimensions of nurses’ perceptions of work environment: (i) Manager Support (support by their supervisor, eight items), (ii) Peer Support (support of nurses among themselves, six items), (iii) Unit Support (support from the supplies, resources, and services they need, six items) (iv) Workload (perceptions on workload, six items), and (v) Intent to Stay (it reflexes the outcome of nurses’ workload perception and measures the tendency to stay at work, five items). The total score range between 29 and 145. Four states (11, 13, 22, and 23) are scored inversely. The higher the score, the more positive the perceptions. The sum of the states provides the overall nursing satisfaction score. It has been used in several studies [19,20] among nurses and has been translated into the Taiwanese language [21] with very good reliability. Also, demographic and job characteristics (gender, age, and rotating shifts) were collected.

The translation process

Τhe procedure of translation was carried out according to guidelines of the World Health Organization (WHO) [22]. First, the forward translation was carried out. Particularly, two Greek head nurses, who were bilingual and connoisseurs of the English culture, translated the English version of the IWPS-R into the Greek language. Second, the lead researcher of this study compared the two Greek versions, and a different Greek version of the scale was produced (first reconciliation version). The next step included the backward translation. The Greek version was translated into the original language (English) by an independent bilingual translator (who had not read the original version), and a new version was produced (second reconciliation version).

The cultural adaptation process

The second reconciliation version was provided to 20 nurses. According to the WHO guidelines [22], at least 10 participants are required for the cultural adaptation. Nurses read the questionnaire and were asked by the researchers if the questionnaire is understandable (cognitive interview process). In case of unclear and difficult points, researchers asked nurses to give an alternative wording without changing the meaning (cognitive debriefing review). Their proposals were integrated into the second reconciliation version, and thus the final Greek version emerged. Then, a General Impressions Tool was provided to the nurses asking them if the questionnaire is understandable, if there are unknown words, and if they chose any alternative word or expression. Most of the nurses (n = 18, 90%) answered that there are no unknown words and the questionnaire is understandable, while only 10% answered that they had difficulties.

Reliability

The reliability was tested through the assessment of stability (test-retest reliability method) and homogeneity (internal consistency reliability via Cronbach's alpha index). For the test-retest reliability, the questionnaire was provided to 40 nurses twice over a two-week interval (test-retest method). The interval of two weeks guarantees that the participants cannot remember the previous answers [23]. For the internal consistency reliability, Cronbach's alpha index was used.

Data analysis

Quantitative variables are presented as mean values (±standard deviation) and qualitative variables as absolute and relative frequencies. The intraclass correlation coefficient (ICC) was performed to test the stability between the two measurements. The Cronbach’s alpha index was applied to investigate the internal consistency reliability of the scale (accepted values > 0.70) [24], while for the study of the construct validity, exploratory factor analysis was performed through the axis rotation method: Varimax with Kaiser normalization. The accepted value of each item loading is > 40 [25]. The criterion of the convergent validity was between the five dimensions of the scale. If the correlation coefficient (r) ranges from 0.1 to 0.3, the correlation is assumed to be low, 0.31 to 0.5 - moderate, and > 0.5 - high [26]. The statistical level was set at <0.05 as it indicates statistical significance. All analyzes were performed with the Statistical Package for the Social Sciences (SPSS), version 21.0 (IBM Corp., Armonk, NY).

Ethics

To carry out the study, licenses were secured from the scientific councils of a general hospital (approval number: 23, 10/02/2021) and a psychiatric hospital in Athens (approval number: 34, 24/02/2021). In all cases, the participants were informed orally and in writing about the aims and objectives of the work, the confidentiality and anonymity of the answers, their right to withdraw at any time during the procedure, and that data will not be shared with anyone. After this, the written consent form was signed by all nurses.

## Results

The participants’ demographic characteristics are presented in Table 1. The mean age was 45 years old (SD ± 8.3); 61.3% have rotating shifts, while the total working experience as nurses was 17.5 years (SD ± 8.2).

**Table 1:** Demographic characteristics of participants (N = 365) SD, Standard deviation.

Demographic Characteristics	Frequency	%
Gender		
Female	287	78.6
Male	78	21.4
Educational level		
High school	167	54.2
University	198	45.8
Marital status		
Married	212	58.1
Unmarried	115	31.5
Divorced	33	9
Widowed	5	1.4
Working department		
Internal medicine sector	66	18.1
Surgical sector	38	10.4
Intensive care unit	30	8.2
Psychiatric	135	37
Other	96	26.3
Rotating shifts		
Yes	220	61.3
No	145	39.7
	Mean (SD)	Min-Max
Age (years)	45(8.3)	25-67
Total working experience as a nurse (years)	17.5 (8.2)	1-35

To study the structural validity of the scale, exploratory and confirmatory factor analyses were carried out. The value of the Kaiser-Meyer-Olkin index for the adequacy of the sample (0.869) and Bartlett's test of sphericity (x2(406), p < 0.001) showed that the sample is adequate; therefore, factor analysis will reveal sufficient results. From the exploratory factor analysis, five factors were emerged: Manager Support (Items 1, 5, 9, 14, 20, 21, 24, 27); Peer Support (Items 7, 8, 15, 16, 26, 29); Unit Support (Items 6, 10, 12, 18, 19, 25); Workload (Items 2, 3, 4, 11, 22, 28); and Intent to Stay (Items 11, 13, 17, 23, 28). These factors explain 51.4% of the total variance (Kaiser criterion, eigenvalue > 1). The values of items’ loadings were ranged from 0.416 (item 26) to 0.862 (item 12) (Table 2).

**Table 2:** Item loadings in factor analysis of the IWPS-R Total variance: 51.4%; Kaiser-Meyer-Olkin measure of sampling adequacy: 0.869; Bartlett's test of sphericity: x2(406), p < 0.001; extraction method: principal component analysis; rotation method: Varimax with Kaiser normalization. *Dual loadings. IWPS-R, Individual Workload Perceptions Scale-Revised.

Items	Manager Support	Peer Support	Unit Support	Workload	Intent to Stay
1	0.592				
2				0.681	
3				0.653	
4				0.483	
5	0.644				
6			0.681		
7		0.668			
8		0.753			
9	0.483				
10			0.674		
11				0.548*	0.417*
12			0.862		
13					0.671
14	0.671				
15		0.714			
16		0.630			
17					0.707
18			0.646		
19			0.674		
20	0.750				
21	0.687				
22				0.738	
23					0.826
24	0.717				
25			0.418		
26		0.416			
27	0.623				
28				0.415*	0.679*
29		0.760			
Eigenvalues	7.787	2.345	1.896	1.586	1.091
% Variance explained	26.853	8.087	6.539	5.471	4.452

Regarding confirmatory factors analysis (CFA), the model tested was equivalent to the original factorial structure of the IWPS-R as proposed by the authors. As suggested in Table 3, this model presented a reasonably good fit to the data. Tucker-Lewis index (TLI) was higher than 0.8 and lower than 0.9; comparative fit index (CFI) was close to 0.9, and root mean square error of approximation (RMSEA) was 0.064 and lower than 0.10. Overall, our CFA confirmed the five-dimensional structure of IWPS-R.

**Table 3:** Model fit for the five-factor model of the IWPS-R for the Greek sample (N = 365) TLI, Tucker–Lewis index; CFI, confirmatory fit index; RMSEA, root mean square error of approximation; IWPS-R, Individual Workload Perceptions Scale-Revised.

TLI	CFI	RMSEA
0.836	0.851	0.064

As far as convergent validity is concerned, a correlation was conducted between the factors of the IWPS-R through the coefficient r. There is a positive correlation between all the domains of the scale (p < 0.001). The highest value of r is 0.794, while the lowest value is 0.320 (Table 4).

**Table 4:** Correlation between factors of IWPS-R *Correlation is significant at the 0.01 level (two-tailed). IWPS-R, Individual Workload Perceptions Scale-Revised.

	Manager Support	Peer Support	Unit Support	Workload	Intent to Stay
Manager Support					
Peer Support	0.550^*^				
Unit Support	0.460^*^	0.461^*^			
Workload	0.501^*^	0.463^*^	0.369^*^		
Intent to Stay	0.411^*^	0.320^*^	0.241^*^	0.579^*^	
Overall Nurse Satisfaction	0.778^*^	0.734^*^	0.653^*^	0.794^*^	0.733^*^

Between the first and the second measurements, no significant differences were shown as the ICC, both in the total score and items separately (ICC = 0.990, p < 0.001), revealed a strong correlation between the two measurements. Also, the r values ranging from 0.82 to 0.92 (p < 0.001) support the excellent correlation and, therefore, the stability of the scale (Table 5).

**Table 5:** Test-retest reliability of the IWPS-R (N = 45) P < 0.001; SD, standard deviation; ICC, intraclass correlation coefficient; IWPS-R, Individual Workload Perceptions Scale-Revised.

IWPS-R Factors	Mean (±SD) 1^st^ Measurement	Mean (±SD) 2^nd^ Measurement	Pearson’s r Correlation	ICC
Manager Support	2.94 (1.20)	2.98 (1.16)	0.92	0.95
Peer Support	3.20 (1.0)	2.96 (0.99)	0.87	0.94
Unit Support	3.33 (1.0)	3.27 (1.34)	0.91	0.95
Workload	3.51 (1.1)	3.41 (1.16)	0.84	0.91
Intent to Stay	3.61 (1.1)	3.65 (1.22)	0.83	0.95
Total	3.58 (1.5)	3.39 (15.2)	0.82	0.91

To test the internal consistency reliability of the IWPS-R, Cronbach’s alpha index was used. According to the results, the total Cronbach’s alpha was found to be 0.878, while the Cronbach’s alpha indexes of each factor ranged between 0.69 and 0.86. The above values indicate the very good internal consistency of the scale. In addition, if an item was deleted, Cronbach’s alpha index would not increase. In Table 6, the descriptive statistics and the Cronbach’s alpha values of the scale are presented. The dimension “Manager Support” had the higher Cronbach’s alpha value. The total score of the scale was 3.65 (SD ± 0.50). The dimension “Peer Support” showed the highest value (3.90), while the lowest value (3.39) is pointed out in “Unit Support.”

**Table 6:** Scales descriptive statistics and internal consistency reliability of IWPS-R (N = 365) SD, Standard deviation; IWPS-R, Individual Workload Perceptions Scale-Revised.

	Min-Max	Mean	SD	Cronbach’s Alpha Values
Manager Support	1.38–5.00	3.89	0.66	0.860
Peer Support	1.67–5.00	3.90	0.64	0.797
Unit Support	1.33–5.00	3.39	0.60	0.692
Workload	1.33–5.00	3.46	0.67	0.690
Intent to Stay	1.00–5.00	3.61	0.85	0.772
Overall Nurses' Satisfaction Score	1.58–4.81	3.65	0.50	0.878

## Discussion

This study was conducted in two large hospitals in the broad area of Athens city aiming to study the psychometric properties of the IWPS-R. The scale was used for the first time among Greek nurses. The importance of the study is great as the knowledge and understanding of the perceived work environment can lead to a better understanding of the problems faced by nurses in the practice of the nursing profession, better staffing, and support from management, therefore leading to the improvement of the quality of care provided [27].

The results of the current study indicate that the Greek version of the IWPS-R is a validated and reliable scale. The revised IWPS has not been weighted in other cultures other than the Taiwanese (T-IWPS-R) [21]. For this reason, there are not enough validity and reliability studies to compare the results.

According to the results of the research, the Greek version of the scale did not create particular problems for the participants as it was understandable and simple without medical terminology. The translation and cultural adaptation of a questionnaire is not a simple translation of the words but it presupposes the adaptation of the scale to the particular cultural elements of each country. This was achieved through the method of forward and backward translation by four independent translators. These translators were specialists (nurses) and spoke English fluently.

The results of the reliability and internal coherence of the scale indicate that the scale is a reliable tool for measuring the nurses’ views on the work environment. In the repeatability test, no significant changes in ICC were observed between the first and second measurements indicating that the characteristic being measured remains the same [28]. Regarding the internal consistency of the IWPS-R, the total Cronbach’s alpha index was found to be 0.878, which indicates excellent internal consistency. The subscale “Workload” had the lower Cronbach’s alpha (0.69), while “Manager Support” had the highest (0.860). Comparing these values with the Cronbach’s alpha value of the Taiwanese version of the scale [21], we conclude that Cronbach’s alpha is rated at similar levels as the lowest index is observed in the factor “Workload” and the highest is observed in “Manager Support.” Also, if an item was removed from the scale, there would be no increase in the Cronbach’s alpha index. This finding means that all items had significant internal coherence with each other.

The construct validity was investigated through the exploratory factor analysis. The dimensions that emerged in this study were “Manager Support,” “Peer Support,” “Unit Support,” “Workload,” and “Intent to Stay.” Similar results are reported in a previous study [18] among perianesthesia nurses of the American Society of PeriAnesthesia Nurses (ASPAN) and the study of psychometric properties of the T-IWPS-R [21]. At this point, it should be noted that all questions remained on the scale supporting the previous study [13] in which the original scale was revised and in contrast to the validation study of the Taiwanese version [21] where five items were removed.

The exploratory factor analysis provided a five-factor model solution explaining 51.4% of the total variance. The factor “Manager Support” had the highest variance (26.853), while the lowest explained variance (4.452) was observed in the factor “Intent to Stay.” The high variance value of the “Manager Support” indicates the great influence of the managers on the nursing working environment in Greece. This finding reflects the results of Lin et al. who investigated the construct validity of the T-IWPS-R, both regarding the highest and the lowest variances [21].

Regarding the loadings of the items, it should be highlighted that two items (item 11, “My current workload will cause me to look for a new position” and item 28 “My current work environment makes me want to stay and work here”) were charged into two factors (“Workload” and “Intent to Stay”) just as in the revision study of Cox et al. [13]. In general, comparing the factor analysis of this study with the structure of the IWPS-R and T-IWPS-R, we could conclude that no particular conceptual differences appear between the three versions.

A correlation between the factors that emerged was carried out to investigate the convergent validity of the Greek IWPS-R. The positive relationship between the dimensions supports the convergent validity of the Greek version of the scale.

Strengths and limitations

A strength of this study is the rare attempt to translate and weigh the IWPS-R in a European country as the IWPS-R has been weighted only for Taiwanese nurses. Also, the sample comes from two very large hospitals of Athens where patients from all over the capitals and areas of Western Greece are treated. As the limitations, it should be considered that the study was conducted during the pandemic COVID-19, so the answers may be affected by the working conditions.

## Conclusions

The psychometric properties of the Greek version of the IWPS-R are very good supporting that the scale is a reliable and valid tool to measure the nurses’ perceptions of the work environment. It is a short tool, which can be completed in minutes and easily evaluated, thus allowing supervisors and managers to easily measure and evaluate the work environment and quickly evaluate their employees by incorporating appropriate interventions to provide the best care. In addition, managers may provide the conditions (emotional and material support) to create a suitable environment with less workload for nurses and better quality of patient care. Also, it is necessary to administer the tool to other hospitals to strengthen its psychometric properties. The intercultural study of the working environment is proposed in order to identify the cultural differences between the United States, Taiwan, and Greece.
